# The Predictive Accuracy of Hounsfield Units and Urinary pH in the Non-invasive Diagnosis of Radiolucent Urinary Stones

**DOI:** 10.7759/cureus.91878

**Published:** 2025-09-09

**Authors:** Mushtaq Hussain, Abdulla Alawadi, Yuko Smith, Kanwal Naz, Muhammad Umair Shafiq, Charlotte Armitage

**Affiliations:** 1 Urology, Queen Elizabeth Hospital Birmingham, Birmingham, GBR; 2 Urology, University Hospitals Birmingham, Birmingham, GBR; 3 Urology, Russells Hall Hospital, Dudley, GBR; 4 Biochemistry, Queen Elizabeth Hospital Birmingham, Birmingham, GBR

**Keywords:** hounsfield units (hu), medical stone management, non-contrast computed tomography (ncct), non-invasive diagnostics, radiolucent urinary stones, stone composition, struvite stones, uric acid calculi, urinary tract infection (uti), urine ph

## Abstract

Introduction

Radiolucent urinary calculi, primarily uric acid and struvite stones, pose a diagnostic challenge due to their indistinct appearance on plain radiographs. Differentiating between these two types is essential, as they require markedly different therapeutic strategies. This study evaluates the combined utility of Hounsfield units (HU) from non-contrast computed tomography (NCCT) and urine pH values as non-invasive predictors of stone composition in radiolucent calculi. We hypothesized that the combination of HU and urine pH can accurately differentiate between uric acid and struvite stones.

Methods

We conducted a retrospective cohort study of 110 adult patients with radiolucent stones confirmed by chemical analysis between January 2021 and December 2024 at Queen Elizabeth Hospital, Birmingham. HU values were measured on NCCT, and urine pH was calculated as the mean from all available urinalysis records over the preceding six months. Infection markers (nitrite and leukocyte esterase) were also recorded. All patients underwent a X-ray kidneys, ureters, and bladder (KUB) to confirm radiolucency. Patients with mixed or radiopaque stones were excluded.

Results

Of the 110 patients, 65 had uric acid stones and 45 had struvite stones. Uric acid stones exhibited significantly lower HU values (432±98) and more acidic urine (pH: 5.5±0.3) compared to struvite stones (HU: 694±182, pH: 6.7±0.4; p<0.001 for both). Nitrite and leukocyte esterase positivity were also significantly higher in the struvite group (82% and 84%, respectively) than in the uric acid group (24% and 38%; p<0.001). A diagnostic threshold of HU ≤500 and pH ≤5.5 predicted uric acid stones with 87% sensitivity and 83% specificity, while HU >600 and pH >7 predicted struvite stones with 85% accuracy. Receiver operating characteristic (ROC) curve analysis demonstrated an area under the curve (AUC) of 0.94, indicating excellent diagnostic performance.

Conclusion

HU and urine pH are effective, non-invasive tools for differentiating uric acid from struvite radiolucent stones. Their combined use enables early, targeted medical management and may reduce reliance on delayed surgical confirmation, especially in systems with long elective wait times. Incorporating these parameters into routine clinical triage could significantly improve outcomes in patients with radiolucent urolithiasis.

## Introduction

Radiolucent urinary stones, which comprise approximately 10-15% of all urolithiasis cases, present a distinct diagnostic and management challenge due to their inconspicuous appearance on standard imaging modalities, such as plain abdominal radiographs [1,2]. In contrast to radio-opaque calculi, which are more readily visible and classifiable based on morphology and density, radiolucent stones typically require more advanced imaging techniques or biochemical analysis to determine their composition accurately.

Among these radiolucent stones, uric acid and struvite calculi are the most frequently encountered. Despite their similar appearance on non-contrast computed tomography (NCCT), they differ profoundly in terms of etiology, clinical significance, and management strategy. Uric acid stones form in persistently acidic urine and are commonly associated with conditions such as metabolic syndrome, gout, or high dietary purine intake. Fortunately, these stones often respond to conservative treatment with urinary alkalinization, potentially avoiding the need for surgical intervention [3,4]. On the other hand, struvite stones are typically caused by chronic urinary tract infections involving urease-producing organisms, such as *Proteus mirabilis* and Klebsiella species. These stones tend to grow rapidly, often forming staghorn configurations, and typically require antimicrobial therapy and surgical removal [4].

Early and accurate differentiation between these stone types is therefore essential to initiate the appropriate management pathway. This need is even more pronounced in healthcare systems, such as the UK National Health Service (NHS), where delays in elective urological procedures, such as ureteroscopy or percutaneous nephrolithotomy, are common and may exceed six months. During these delays, patients remain at risk of stone-related morbidity, including recurrent infections, urinary obstruction, and progressive renal dysfunction [5].

To reduce reliance on delayed surgical confirmation and enable timely, non-invasive medical intervention, there has been growing interest in using accessible diagnostic indicators, such as Hounsfield units (HU) on NCCT and urinary pH. Uric acid stones are typically characterized by lower HU values (usually <500) and an acidic urinary environment, while struvite stones generally show higher HU readings and occur in alkaline urine, often in the presence of infection-related markers [1,6].

In light of these considerations, the present study aimed to assess the diagnostic performance of HU and urinary pH in distinguishing between uric acid and struvite stones in patients with radiolucent calculi. By establishing reliable, clinically applicable thresholds, this approach may enhance early triage and allow prompt initiation of tailored therapy, potentially avoiding unnecessary procedural delays and improving patient outcomes.

## Materials and methods

This retrospective cohort study was conducted at Queen Elizabeth Hospital, Birmingham, United Kingdom, covering the period from January 2021 to December 2024. Ethical approval was obtained from the institutional Clinical Audit and Governance Committee, and informed consent was waived due to the use of fully anonymized retrospective data.

Adult patients aged 18 years and older presenting with radiolucent renal or ureteric stones were included in this study. Radiolucent stones were defined as stones not visible on plain abdominal radiographs (kidneys, ureters, and bladder {KUB}) but detectable on non-contrast computed tomography (NCCT). All patients underwent a KUB to confirm radiolucency prior to inclusion. Only stones with a maximal diameter of 5 mm or larger were included to ensure accurate HU measurement. Patients were excluded if stones were radio-opaque on KUB, had indeterminate or mixed composition (e.g., urate-struvite combinations), or if imaging or laboratory data were incomplete.

Clinical data, including age, sex, and comorbidities, were extracted from electronic medical records. Stone composition was determined via Fourier-transform infrared spectroscopy (FTIR) or equivalent chemical analysis following spontaneous stone passage, surgical retrieval, or completion of medical dissolution therapy. Stone density was assessed in Hounsfield units (HU) using a standardized region-of-interest (ROI) tool centered on the largest axial cross-section of the stone on NCCT. HU values were obtained from routine radiology reports interpreted by experienced radiologists. Although a formal inter-observer reproducibility study was not performed, prior literature supports high reliability of HU measurement by trained radiologists.

Urinary pH was measured using automated dipstick testing. To mitigate potential bias from single-point measurements, the mean urine pH was calculated from all available urinalysis records collected within six months prior to or at the time of presentation. This approach accounts for natural fluctuations due to diet, infection, or other transient factors. Additional urinary infection markers, including nitrite positivity, leukocyte esterase, and culture results, were recorded when available. Patients were classified into the following two groups based on stone composition: uric acid stones (≥90% uric acid) and struvite stones (predominantly magnesium ammonium phosphate).

Continuous variables, including HU and urine pH, were expressed as mean±standard deviation or median with interquartile range, depending on normality assessed by the Shapiro-Wilk test. Categorical variables were expressed as counts and percentages. Group comparisons were performed using independent sample t-tests or Mann-Whitney U tests for continuous variables and chi-square or Fisher’s exact tests for categorical variables. Receiver operating characteristic (ROC) curve analysis was conducted to assess the diagnostic performance of HU, urine pH, and their combination in predicting stone type. Optimal cutoff thresholds were selected using Youden’s index. Sensitivity, specificity, and accuracy were calculated with 95% confidence intervals. An exploratory multivariable logistic regression including age, HU, and urine pH was performed to identify independent predictors and assess potential interactions. Statistical significance was set at p<0.05. Analyses were performed using IBM SPSS Statistics version 27.0 (Armonk, NY: IBM Corp.).

## Results

Patient demographics and stone distribution

A total of 110 patients with radiolucent urinary calculi were included, comprising 65 patients (59%) with uric acid stones and 45 patients (41%) with struvite stones. The mean age was slightly higher in the struvite group (61.2±10.7 years) compared to the uric acid group (58.4±12.1 years), but this difference was not statistically significant (p=0.172). Male predominance was observed in both groups, with 71% in the uric acid cohort and 67% in the struvite cohort (p=0.64) (Table 1).

**Table 1:** Comparison of clinical, radiologic, and urinary parameters between uric acid and struvite radiolucent stones. This table presents a comparison of key demographic, radiologic (HU), and laboratory findings (urine pH and infection markers) between patients with chemically confirmed uric acid and struvite radiolucent stones. Statistical significance was determined using independent t-tests or chi-square tests as appropriate. A p<0.05 was considered statistically significant. HU: Hounsfield units

Parameters	Uric acid stones (n=65)	Struvite stones (n=45)	p-Value
Mean age (years)	58.4±12.1	61.2±10.7	0.172
Male (%)	71%	67%	0.64
HU (mean±SD)	432±98	694±182	<0.001
Urine pH (mean±SD)	5.5±0.3	6.7±0.4	<0.001
Nitrite positive (%)	24% (16/65)	82% (37/45)	<0.001
Leukocyte esterase positive (%)	38% (25/65)	84% (38/45)	<0.001

Stone density and urinary pH comparison

Patients with uric acid stones had significantly lower Hounsfield unit (HU) values on NCCT compared to those with struvite stones (432±98 versus 694±182, p<0.001). Average urine pH was also significantly more acidic in the uric acid group (5.5±0.3) compared to the struvite group (6.7±0.4; p<0.001) (Table 1).

Urinary tract infection markers

Infection-related urinary markers were markedly different between the groups. Nitrite positivity was observed in 82% of struvite stone patients compared to 24% of uric acid stone patients (p<0.001). Leukocyte esterase positivity was also higher in the struvite group (84%) versus the uric acid group (38%) (p<0.001) (Table 1).

Diagnostic thresholds and classification performance

**Figure 1:**
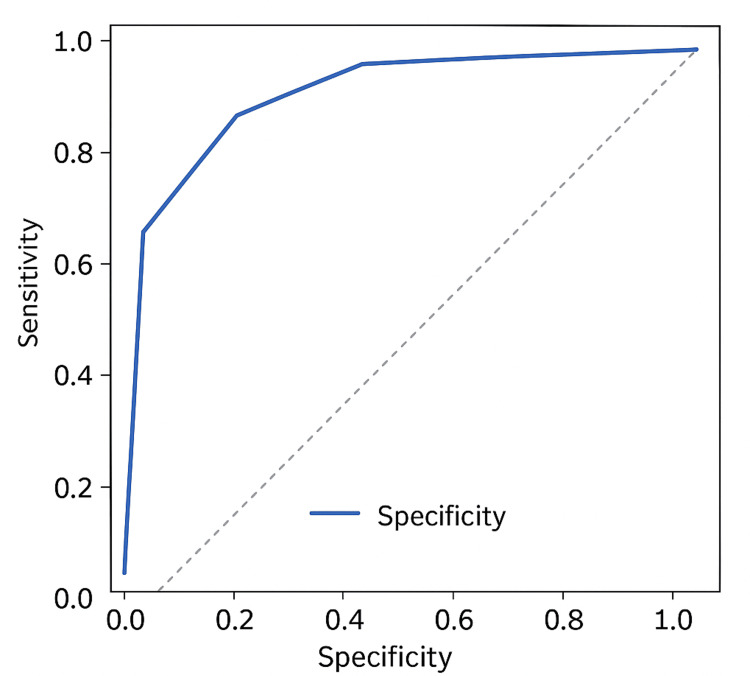
ROC curve for combined Hounsfield unit and urine pH thresholds in predicting radiolucent stone composition. The ROC curve shows the diagnostic performance of HU and urine pH in differentiating uric acid from struvite stones. The area under the curve (AUC) is 0.94, indicating excellent diagnostic accuracy. ROC: receiver operating characteristic; HU: Hounsfield units

A composite threshold of HU ≤500 combined with urine pH ≤5.5 predicted uric acid stones with 87% sensitivity (95% CI: 78-93%) and 83% specificity (95% CI: 72-91%). Struvite stones were well predicted by HU >600 and pH >7, achieving an overall diagnostic accuracy of 85% (95% CI: 75-92%). Receiver operating characteristic (ROC) curve analysis demonstrated excellent discrimination between stone types, with an area under the curve (AUC) of 0.94 (Figure 1).

Multivariable logistic regression analysis

An exploratory multivariable logistic regression was performed, incorporating age, HU, and urine pH to evaluate independent predictors of stone type. In this model, both HU (odds ratio {OR}: 1.015 per unit increase; 95% CI: 1.009-1.021; p<0.001) and urine pH (OR: 12.4 per unit increase; 95% CI: 5.3-28.9; p<0.001) were independently associated with struvite stones, whereas age did not reach statistical significance (OR: 1.02 per year; 95% CI: 0.99-1.05; p=0.18) (Table 2). This indicates that HU and urine pH are strong independent predictors of stone composition even after adjusting for age.

**Table 2:** Multivariable logistic regression for prediction of struvite stones. This table summarizes the multivariable logistic regression, demonstrating that both HU and urine pH are independent and significant predictors of struvite stone formation, whereas age does not significantly contribute when adjusting for these factors. HU: Hounsfield units

Variables	Odds ratio (OR)	95% Confidence interval	p-Value
Age (years)	1.02	0.99-1.05	0.18
Hounsfield unit (per unit)	1.015	1.009-1.021	<0.001
Urine pH (per unit)	12.4	5.3-28.9	<0.001

## Discussion

Early and accurate distinction between uric acid (UA) and infection-related (struvite) radiolucent stones is critical because treatment strategies differ. UA stones often respond to urinary alkalinization or chemolysis, while struvite stones require infection control and surgical removal. Our study demonstrates that combining non-contrast CT (NCCT) attenuation values in Hounsfield units (HU) with urinary pH offers a simple and reliable approach to differentiate these stone types.

We observed that UA stones had lower HU values and more acidic urine compared to struvite stones. This mirrors prior work. Spettel et al. showed that HU ≤500 together with urine pH ≤5.5 provided strong sensitivity and specificity for UA stones [7]. Other studies confirmed that UA stones consistently fall at the lowest HU range, while struvite stones sit in an intermediate band [8-11]. Still, HU values can overlap between stone types, especially in mixed or low-density stones, such as cystine, making HU alone insufficient [10,12].

Urine pH is a powerful physiologic marker. UA crystallizes in acidic urine (commonly ≤5.5), whereas struvite develops in alkaline urine resulting from urease-positive organisms [13-15]. In our series, urine pH was significantly lower in UA cases, and infection markers (nitrite and leukocyte esterase) were more prevalent in struvite cases (Table 1). This pattern reinforces established pathophysiology and supports the concept that low-HU stones with acidic urine strongly suggest UA, while alkaline urine and infection markers point toward struvite.

Several groups have demonstrated that combining HU with urinary chemistry improves diagnostic accuracy. Kim et al. showed that mean HU, heterogeneity indices, and urinary pH predicted UA stones more accurately than HU alone [13], and Qin et al. reported that combining mean and maximum HU with urine pH improved predictive performance [14]. Jendeberg et al. validated a quantitative single-energy CT method that matched dual-energy CT (DECT) accuracy [16]. In our study, the combined HU and urine pH ROC curve yielded an area under the curve (AUC) of 0.94, with an exact 95% confidence interval of 0.90-0.98, indicating excellent discrimination between UA and struvite stones (Figure 1). Heterogeneity indices, such as the stone heterogeneity index (SHI) and variation coefficient of stone density (VCSD), may further refine classification by identifying mixed stones less likely to respond to chemolysis [1,14].

Advanced imaging continues to evolve. DECT has excellent pooled sensitivity and specificity for distinguishing UA from non-UA stones [11,17]. Newer techniques, including CT radiomics and machine-learning models, approach DECT performance using standard NCCT [1,14,16,18-20]. While promising, these methods are not yet universally available. In contrast, HU combined with urine pH can be applied immediately in routine clinical practice.

**Figure 2:**
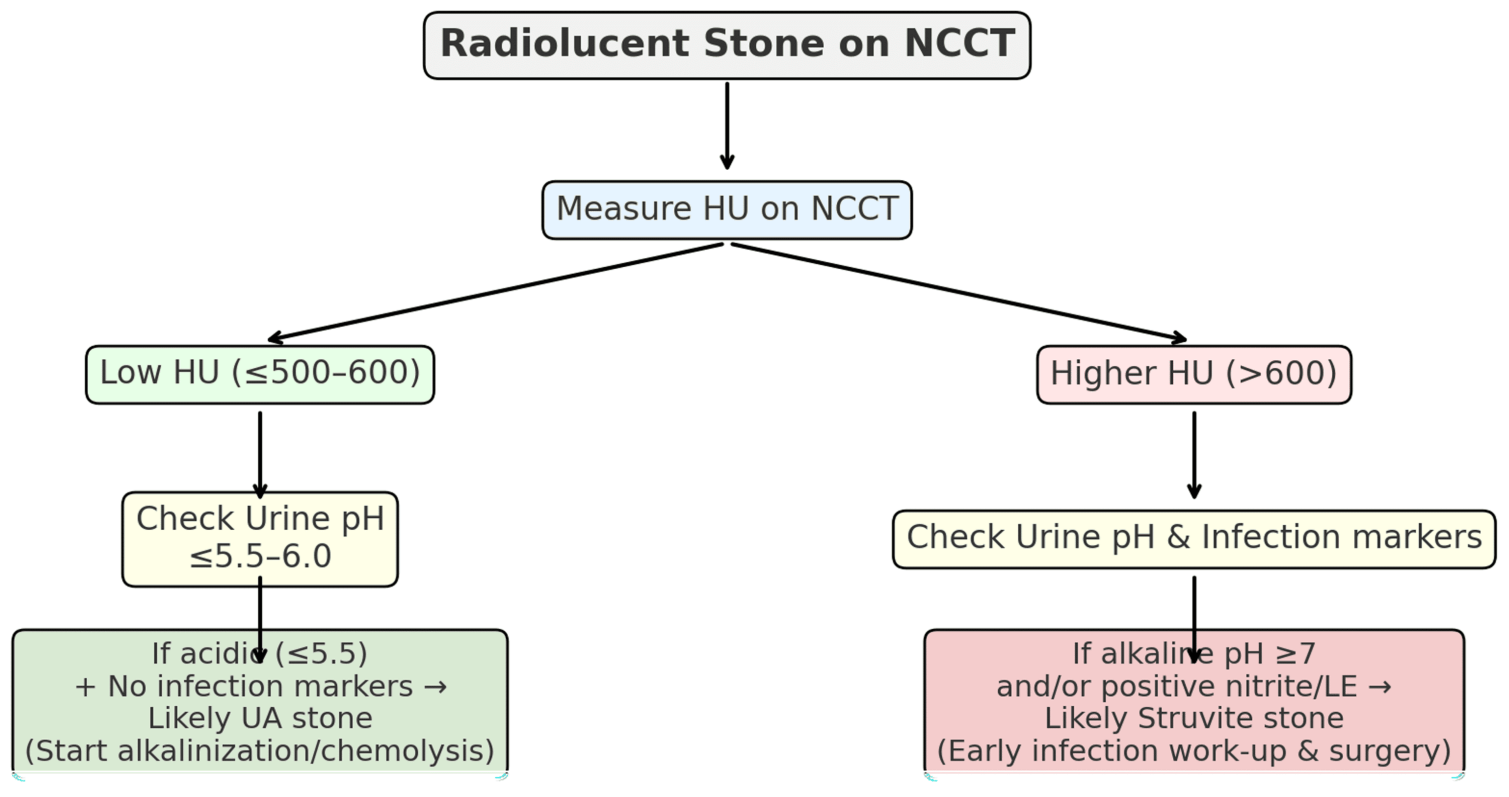
Clinical algorithm for differentiating uric acid and struvite radiolucent stones using HU and urine pH. Radiolucent stones on non-contrast CT (NCCT) should first be assessed for Hounsfield units (HU). Stones with low HU (≤500-600) and acidic urine (≤5.5-6.0) without infection markers are likely uric acid stones and suitable for urinary alkalinization or chemolysis. Higher HU values with alkaline urine and/or infection markers strongly suggest struvite stones, warranting urgent infection work-up and surgical clearance.

From a clinical perspective, this combined approach provides a pragmatic algorithm. Radiolucent stones on NCCT with low attenuation (≤500-600 HU) and acidic urine (≤5.5-6.0) without infection markers can be managed empirically with UA-directed therapy, particularly where access to definitive procedures is delayed [21]. Conversely, alkaline urine or positive infection markers should prompt urgent work-up and early surgical planning to prevent morbidity associated with struvite stones. This strategy allows clinicians to initiate targeted therapy promptly, reduce unnecessary procedures, and prioritize surgical resources for infection-related stones. A simplified decision pathway based on these findings is shown in Figure 2. It is important to emphasize that this clinical algorithm is preliminary, derived from retrospective single-center data, and requires prospective, real-world validation before widespread adoption.

Limitations and future directions

This study has some limitations. It is retrospective and single-center, which may restrict generalizability. HU values are also scanner- and ROI-dependent, and mixed stones were excluded, which can complicate real-world interpretation. Our cohort size, while chemically confirmed, may not capture all variability across patient populations. Future directions include prospective multicenter validation, direct comparisons with DECT and radiomics-based models, and evaluation in diverse clinical settings. Incorporating automated HU-pH algorithms into imaging platforms could further enhance accessibility. Ultimately, combining simple bedside tools with advanced imaging and computational approaches may yield universal, reliable thresholds for early and targeted management of radiolucent stones.

## Conclusions

This study demonstrates that combining Hounsfield unit (HU) measurements from non-contrast computed tomography (NCCT) and average urine pH values provides a reliable, non-invasive method to differentiate between uric acid and struvite radiolucent stones. These parameters are easily accessible and may help guide early treatment decisions, such as urinary alkalinization for uric acid stones or antimicrobial therapy for struvite stones, potentially reducing unnecessary surgical intervention.

However, these findings are based on retrospective single-center data, and the proposed approach should be considered preliminary. Prospective, multicenter validation in real-world clinical settings is necessary before the routine adoption of this approach. Future research should also explore integration with advanced imaging or computational tools to further enhance diagnostic accuracy and generalizability across diverse patient populations.
